# Hazards Associated with the Combined Application of Fungicides and Poultry Litter in Agricultural Areas

**DOI:** 10.3390/jox14010007

**Published:** 2024-01-09

**Authors:** Dario Corrêa-Junior, Cláudio Ernesto Taveira Parente, Susana Frases

**Affiliations:** 1 Laboratório de Biofísica de Fungos, Instituto de Biofísica Carlos Chagas Filho, Universidade Federal do Rio de Janeiro, Cidade Universitária, Ilha do Fundão, Rio de Janeiro CEP 21941-902, Brazil; dariojunior@biof.ufrj.br; 2 Laboratório de Radioisótopos Eduardo Penna Franca, Instituto de Biofísica Carlos Chagas Filho, Universidade Federal do Rio de Janeiro, Av. Carlos Chagas Filho s/n, Bloco G0, Sala 60, Subsolo, Rio de Janeiro CEP 21941-902, Brazil; cparente@biof.ufrj.br; 3 Rede Micologia RJ, FAPERJ, Rio de Janeiro CEP 21941-902, Brazil

**Keywords:** poultry farming, global food demand, environmental challenges, fungal roles, one health

## Abstract

In recent decades, the poultry farming industry has assumed a pivotal role in meeting the global demand for affordable animal proteins. While poultry farming makes a substantial contribution to food security and nutrition, it also presents environmental and public health challenges. The use of poultry litter as fertilizer for agricultural soils raises concerns about the transfer of pathogens and drug-resistant microorganisms from poultry farms to crop production areas. On the other hand, according to the Food and Agriculture Organization of the United Nations (FAO), fungicides represent the second most used chemical group in agricultural practices. In this context, agricultural soils receive the application of both poultry litter as a fertilizer and fungicides used in agricultural production. This practice can result in fungal contamination of the soil and the development of antifungal resistance. This article explores the necessity of monitoring antifungal resistance, particularly in food production areas with co-application of poultry litter and fungicides. It also highlights the role of fungi in ecosystems, decomposition, and mutualistic plant associations. We call for interdisciplinary research to comprehensively understand fungal resistance to fungicides in the environment. This approach seeks to promote sustainability in the realms of human health, agriculture, and the environment, aligning seamlessly with the One Health concept.

## 1. Introduction

Poultry farming plays a pivotal role in meeting the rising demand for affordable animal proteins, particularly in developing nations. Its historical significance in providing consistent protein supplies, contributing to food security, and improving nutrition, especially in rural areas, cannot be overstated. However, this practice is not without its challenges, most notably the environmental and public health concerns it raises [1].

Over the past two decades, global poultry production has witnessed steady growth, and poultry imports are expected to rise even further. In comparison, pork and beef imports are also projected to increase, but poultry remains the preferred choice among consumers [2]. This intensification in poultry production, which often involves large flocks confined in automated systems, gives rise to concerns regarding parasitic infections and zoonotic diseases due to the densely populated living conditions for birds. One notable aspect discussed is the management of poultry litter, which comprises feces, waste, and bedding materials, and its importance in maintaining bird health and crop quality when used as organic soil fertilizer [3].

However, poultry litter can contain pathogens and contaminants, including resistant microorganisms, veterinary drugs, and potential toxic elements. This leads to concerns regarding its safety when used as an organic fertilizer, especially in developing countries with limited data [3,4].

The fungi kingdom is incredibly diverse, estimated to comprise 1.5 to 5 million species. Fungi are vital participants in various ecosystems, contributing to the decomposition of organic matter, forming mutualistic associations with plants, and acting as biocontrol agents. While most fungi play beneficial roles, some are pathogenic to humans, causing diseases that affect millions [5].

The adaptability of fungi to various environmental pressures, including climate change and increased pesticide resistance in agriculture, is noted as a significant area of study. Understanding these adaptations is crucial for developing strategies to manage fungal diseases and preserve ecosystems [2].

In the context of poultry farming, fungi play essential roles in decomposing organic matter in poultry waste, recycling nutrients, and maintaining ecological balance. However, there are certain fungi that can produce mycotoxins, posing health risks when they contaminate poultry feed and food products [6]. Additionally, the article highlights the importance of understanding the fungal microbiota in birds, especially as it relates to the potential transmission of pathogenic microorganisms, including drug-resistant fungi, to humans.

Fungal presence in poultry farms generates organic dust composed of various particles, including fungal spores, some of which can cause allergies, posing health risks to workers [7]. The article also discusses the vital role of fungi in maintaining ecological balance and their interactions with xenobiotics, foreign chemical substances in the body, with applications in bioremediation.

The adverse environmental consequences brought about by fungi, obstructing both soil utilization and animal husbandry for consumption, have driven substantial shifts in the interaction between fungi and their developmental environment [4]. The escalating use of fungicides in poultry farming is a mounting concern, raising the prospect of pathogenic fungi developing resistance due to heightened fungicide consumption. This underscores the critical need for diligent monitoring and effective management of antifungal resistance, including susceptibility testing [8].

In poultry production systems, fungal disease control often focuses on management, hygiene, and prevention practices rather than relying extensively on fungicides. Some typical prevention measures include maintaining a clean, dry environment for birds, rotating grazing areas, and properly disposing of poultry litter [9]. However, it is important to highlight that, even if the use of fungicides in poultry farming is limited, the soils on which poultry litter is applied can receive contamination from pesticides for agricultural use and fungi that have proliferated in the litter. This is because the bedding, which is often made from organic material such as straw, sawdust or rice husks, can contain fungi and pesticide residues that were applied to the growing areas of the litter ingredients. Fungicides are an important class of agrochemicals used to control fungal diseases in crops [10].

The Food and Agriculture Organization of the United Nations (FAO) provides data and information on the use of agrochemicals, including fungicides, in agriculture. These chemicals play a significant role in protecting crops from fungal infections that can cause significant losses in food production. Raising awareness about the responsible use of agrochemicals, including fungicides, is essential to ensure food safety and minimize adverse environmental impacts. Furthermore, strict regulation and monitoring of the use of fungicides in agriculture are essential to ensure that these products are applied safely and effectively, minimizing risks to human health and the environment [11].

This study emphasizes the interconnectedness between poultry litter and fungal ecology in agricultural systems. Poultry litter, a common substrate for poultry, may carry fungal spores and microorganisms from poultry facilities into agricultural soils, affecting biogeochemical cycles and soil microbiology [3]. Additionally, the extensive use of fungicides in crop agriculture raises concerns about their impact on soil, including groundwater contamination and fungal resistance [12]. Investigating the presence and effects of both poultry litter and fungicides in soils is crucial for sustainable agriculture. This study aims to understand the complex relationship between poultry litter and the introduction of fungi from poultry facilities into agricultural soils, as well as the substantial use of fungicides. This research contributes to our knowledge of the environmental and agricultural aspects of poultry litter and fungicide use.

This article sheds light on the urgent need for standardized guidelines and comprehensive research initiatives to effectively address these issues, thereby ensuring food safety and environmental sustainability. The importance of investigating the interaction between poultry farming and fungal ecology cannot be emphasized enough. This focus aligns with the overarching One Health concept, which acknowledges the interconnectedness of human, animal, and environmental well-being. As a result, it calls for collaborative interdisciplinary efforts, the implementation of biosafety measures, and the pursuit of future research endeavors aimed at understanding fungal ecology and tackling associated challenges [2].

The interconnection between environmental sciences, agriculture, microbiology, and public health is highlighted as a crucial aspect for fully understanding the impacts of this complex interaction. However, to further strengthen the work, it is imperative to emphasize specific implementation strategies derived from this interdisciplinary approach. Incorporating collaborative methods such as continuous monitoring, comprehensive data collection, and integrated analysis will provide valuable insights. Furthermore, it is crucial to establish clear expectations regarding expected results, aiming not only to identify possible environmental risks, but also to propose sustainable solutions. By highlighting these specific implementation strategies and anticipated outcomes, the article can catalyze the practical application of interdisciplinary knowledge in promoting environmental and agricultural health. In doing so, it promotes the overarching goal of enhancing human health, fortifying agriculture, and safeguarding environmental welfare.

## 2. Global Poultry Farming

Poultry farming plays an essential role in global food production and has been accompanied by steady growth in poultry production in recent years. This increase is directly associated with the growing demand for affordable animal proteins, particularly in developing countries, where the search for a diversified diet is on the rise [1]. Raising domesticated birds to obtain meat, eggs, and feathers is an ingrained practice in food production, dating back to the beginnings of agriculture. Avian farming stands out for its remarkable efficiency in using a wide range of raw materials, ranging from agricultural and domestic waste to food processing by-products [3]. This approach establishes fowl farming as one of the most effective methods of animal husbandry, ensuring a consistent supply of protein and playing a key role in food and nutrition security, especially in rural areas around the world, with an emphasis on developing countries [1].

Over the two-decade period spanning from 2001 to 2021, there was a consistent annual growth rate of 4 percent in global poultry imports, culminating in a total of 14.2 million metric tons imported by 2021. The United States Department of Agriculture (USDA) anticipates a significant upswing in poultry imports, projecting them to reach 17.5 million metric tons by the year 2031. In contrast, pork imports are also on an upward trajectory, with projections indicating an increase to 14.8 million metric tons by 2031, while beef imports are expected to reach 14.3 million metric tons during the same period [13].

In the FAO’s Food Outlook 2023, the global production of poultry, including chicken, turkey, duck, and goose meats, is expected to increase by approximately 1.35%, reaching a total of 142.6 million tons this year. China is predicted to become the world’s largest producer, surpassing the United States by around 5%, with Brazil ranking third at 11% of the global production. While China and the United States are projected to see modest growth (below half a percent and less than 2%, respectively), Brazil is expected to experience the highest growth rate among the top ten global producers, at 2.74%. Japan is the sole country in this group with a negative outlook, though it signifies a state of relative stability. Approximately 60% of the top ten countries are expected to lose their share of global production, while Brazil stands out with the most significant increase (1.37%) compared to 2022. On a continental scale, Africa is the only region with a potential reduction, and both Asian and European countries are also anticipated to lose their share. Notably, the production of the 27 European Union (EU) member states accounts for approximately 60% of the continent’s total production, with non-member countries, including Russia, Ukraine, and the United Kingdom, responsible for the remaining 40% [14].

Most of the industrial-scale poultry production, chiefly encompassing broilers for meat production and layers for egg production, is carried out on intensive production farms. In this context, flocks ranging from several thousand to hundreds of thousands of birds are maintained, primarily raised in open indoor housing or battery cages equipped with automatic feeding and drinking systems [15]. Recent years have witnessed an intensification of livestock production systems due to an increased demand for animal-derived foods, along with changes in management practices, which have impacted the distribution and intensity of parasitic infections, frequently associated with zoonotic diseases [2,16].

Poultry production is predominantly characterized by densely populated confinement structures for birds. Consequently, maintaining the ideal microclimate and animal hygiene conditions can pose significant challenges. Despite the utilization of mechanical ventilation systems to safeguard bird health, microorganisms found in animal bedding can readily accumulate and disperse in the air. Poultry litter, composed of feces, food waste, bedding materials, and feathers, is highly regarded as a cost-effective, high-quality organic soil fertilizer that contributes to enhancing crop quality and productivity, justifying its extensive global use [17].

Ensuring efficient poultry production requires adequate litter material with appropriate chemical and physical characteristics, along with controlled microbial counts. Wood-based bedding has been associated with improvements in bird performance. Besides its organic composition, the material used as animal bedding can harbor various pathogens, including viruses, bacteria, parasites, and fungi. Furthermore, the combination of bedding materials with chicken droppings and feathers appears to influence pathogen development [6,16].

Microbiological air contamination in poultry facilities, including an analysis of microbial composition, has been the subject of comprehensive studies [2]. Concerns about rising foodborne outbreaks have led to the identification of bacteria in animal bedding, with certain *Salmonella* strains exhibiting antimicrobial resistance [18]. The presence of microbial pathogens is a matter of utmost importance in the food industry; thus, the quality of litter material can directly impact food safety [10].

Brazil and the United States are the primary producers and exporters of poultry meat, with substantial growth expected in the coming years. In the case of Brazil, a 29% increase in production is anticipated by 2028. Concerning egg production, the domestic market constitutes the primary destination, representing nearly 100% of consumption [17]. Despite playing a limited role in exports, Brazil ranks among the largest egg producers globally, with consumption steadily rising over the years. The impacts associated with the growth of the poultry production chain extend beyond production areas and may have regional and global ramifications [17].

It is vital to emphasize that, in addition to export and increased domestic consumption concerns, there are significant challenges pertaining to environmental and public health impacts arising from large-scale production systems. Nonetheless, in Brazil, the discussion of these impacts is still in its early stages, and there is a scarcity of available information, despite the importance of these issues for food security, environmental quality, and public health [18,19,20].

The significant growth seen in global poultry production in recent years has not only boosted the industry but has also raised serious concerns about the environmental impacts associated with this expansion. As we explore the rise of poultry farming on a global scale, it is crucial to take a closer look at the environmental challenges that arise from this rapid expansion.

### Poultry Litter

Poultry litter comprises any material placed on the ground to serve as a bed for birds, receiving excrement, feed remains, feathers, spilled food, medicines, and water. Its primary function is to prevent birds from direct contact with the ground, while absorbing moisture, incorporating waste, promoting the birds’ well-being, and improving their comfort [8]. There are several materials that can be used in the composition of these beds, from the most common, such as rice husks, wood shavings or sawdust, to alternatives such as sugarcane bagasse [3]. The use of chicken litter in the soil as an organic fertilizer represents the most economical and environmentally friendly approach to disposing of this material from the rapidly expanding poultry industry around the world [3].

Nonetheless, a key challenge lies in optimizing the advantages of chicken litter as an organic fertilizer while concurrently mitigating its potential adverse impacts on the environment [8]. Among the foremost concerns related to the safety of poultry litter is its high nutrient content, being notably rich in nitrogen and phosphorus, which has the potential to lead to the contamination of freshwater bodies [3]. Chicken litter carries pathogenic microorganisms, encompassing bacteria, fungi, viruses, parasitic protozoa, and helminths. Additionally, it may contain antibiotics, along with pathogenic microorganisms that harbor antibiotic-resistant genes, heavy metals, sex hormones like estrogen (17β-estradiol and testosterone), polycyclic aromatic hydrocarbons, and persistent organic pollutants such as dioxins, furans, and polychlorinated biphenyls [3].

It is essential to highlight that the presence of contaminants poses a threat to the natural environment and has the potential to undermine the environment’s usability by humans [20]. Furthermore, poultry litter can serve as a reservoir for pathogens, which are organisms capable of inducing diseases in susceptible hosts, encompassing humans, animals, or plants [1]. This regulatory diversity poses challenges in providing farmers with clear guidance on the appropriate and safe application of chicken litter to the soil, particularly for the purpose of restoring productivity to degraded soils [10].

Consequently, it is imperative to conduct more comprehensive studies to evaluate the degree of contamination in poultry litter, both from broilers and layers, with a particular focus on developing countries, where data is limited. Moreover, there is a need to establish standardized guidelines encompassing all contaminants present in chicken litter, with the aim of ensuring consistent practices across all regulatory agencies to facilitate the safe disposal of this material into the soil [2,16].

## 3. Fungi and Poultry Farming

### 3.1. Fungi Kingdom

The “Fungi” kingdom encompasses a remarkable diversity of species, estimated to range from 1.5 to 5 million, with approximately 600,000 of them having already been documented [5]. Fungi play a fundamental role in the ecosystem functions of terrestrial and aquatic ecosystems, performing several essential functions. Fungi play a crucial role in the decomposition of organic matter, an essential process for the recycling of nutrients in ecosystems. They decompose complex organic materials, such as fallen leaves, twigs, and plant debris, transforming them into simpler components. This contributes to nutrient cycling, releasing essential elements back into the soil, making them available to plants [21].

Many fungi establish mutualistic associations with plant roots, known as mycorrhizal symbiosis. In this symbiotic relationship, mycorrhizal fungi help plants absorb nutrients, such as phosphorus and nitrogen, in exchange for carbohydrates and organic compounds provided by plants. This symbiosis benefits both fungi and plants, improving plant nutrition and fungal survival [5]. Some fungi act as biocontrol agents, fighting pests and pathogens that affect plants and animals. These fungi can parasitize or compete with harmful organisms, contributing to the regulation of pest populations and the maintenance of ecological balance [5].

Fungi play an essential role in the production of food and beverages. Yeasts are used in the fermentation of bread, beer, wine, and cheese, while edible mushrooms are cultivated and consumed around the world [21]. Certain fungi could degrade organic pollutants, such as hydrocarbons and toxic compounds. They are used in bioremediation processes to clean up contaminated environments, helping to restore damaged ecosystems [5]. Fungi play an important role in the formation and stabilization of soil. They assist in the decomposition of organic matter, releasing organic acids that contribute to the chemical alteration of soil particles. This influences the texture and water-holding capacity of the soil, impacting plant growth and soil quality [5]. Fungi serve as a food source for various organisms, including insects, nematodes, and vertebrate animals. Furthermore, the process of decomposition of fungi provides food for a variety of invertebrates, contributing to the food chain of ecosystems [5].

Among these species, a few hundred could cause disease in humans. Fungal diseases can be clinically classified as superficial, cutaneous, subcutaneous, and systemic [22]. In recent years, fungal diseases are estimated to have resulted in more than 1.6 million deaths annually, affecting more than a billion people with serious fungal diseases [22]. However, it is important to note that many cases go unreported, which raises doubts about the precision of available estimates [23].

In recent decades, there has been an exponential growth in the knowledge of pathogenic fungi that affect humans, driven by the development and improvement of genetic, genomic, and molecular tools specific to these microorganisms. On the other hand, the development of new medicines has been slow, and technical limitations persist in the clinical diagnosis of fungal diseases. In general, fungal diseases are not notifiable, which raises questions about the reliability of most estimates [23]. Fungal pathogens probably faced environmental pressures over time and, in their evolution, adapted their pathogenic mechanisms mainly to interact with less complex organisms, such as plants, environmental predators, invertebrates, and less complex mammals, such as amphibians and reptiles, before interactions with human beings arose [24]. These interactions often involve accidental hosts. On the other hand, there are several commensal fungi that can cause disease in humans when there is an imbalance in the immune system. For a fungus to cause damage to a human being and thus cause a disease, this microorganism must possess at least a triad of mechanisms: (I) the ability to obtain nutrients to sustain its metabolism, (II) ability to grow at temperatures close to 37 °C and (III) the ability to avoid, escape, or evade the human immune system [25].

Fungi have demonstrated a remarkable ability to adapt to diverse environmental pressures, including climate change, increased atmospheric temperature and changes in the water cycle in different regions of the planet. Furthermore, they face the challenge of increasing resistance to pesticides in agricultural environments. This adaptive flexibility is crucial to the survival of these organisms, allowing them to thrive in challenging environments [26]. Understanding these adaptative mechanisms can be fundamental for the development of effective strategies for controlling and preventing fungal diseases. Additionally, this knowledge also contributes to the preservation of ecosystems where these organisms play vital roles [27].

### 3.2. Fungi in Poultry Farms

In poultry farming, fungi play an important role in the decomposition of organic matter present in poultry waste, such as food remains and bird excrement. They help in recycling nutrients and maintaining ecological balance [7]. However, some species of fungi can produce mycotoxins, substances that are toxic to animals when feed and food are contaminated. Furthermore, these fungi can also be pathogenic and cause fungal infections [28]. On the other hand, yeasts can be used as probiotics, as is the case with *Saccharomyces cerevisiae*, to improve the intestinal health of birds, promoting digestion, increasing nutrient absorption, and improving resistance to infections [29]. The gastrointestinal and reproductive microbiota of birds are composed of bacteria, fungi, viruses, and protists, and are characterized by commensal, symbiotic, and pathogenic relationships with the host [28].

Differences between host and microbiota have been characterized by nutrient exchange, modulation of the immune system, exclusion of pathogens, and gastrointestinal tract and reproductive physiology [3]. The composition and function of the microbiota can be affected by many factors, including host age, genotype and sex, diet composition and form, food additives such as antibiotics, probiotics, prebiotics, postbiotics, symbiotics, phytobiotics, bacteriophages, stress, and the location in the reproductive tract [30]. Although birds are known to harbor pathogens with zoonotic potential, the yeast microbiota of these birds’ gut biomes is still poorly understood [30].

There is growing concern that poultry products may be underappreciated sources of pathogenic microorganisms, especially certain yeast species that may be pathogenic to other species, including humans. These yeasts can behave as pathogens when they find a suitable and conducive host, in addition to developing antimicrobial resistance when moving between different niches [31]. Previous studies have documented the occurrence of different yeast species as natural residents of the avian gut, but the behavior and ecological factors that contribute to the conversion of these organisms from harmless commensals to pathogens are still poorly understood [30].

The state of the host immune system and putative yeast virulence factors play important roles in triggering infections and invasion of host tissues [3]. Studies have reported the presence of medically important yeasts in pigeon feces, confirming that these birds can serve as reservoirs and disseminators of *Cryptococcus* spp. and other yeasts for humans [32]. Furthermore, birds such as parrots have been identified as sources of dissemination of *Trichosporon* spp., *Candida* spp., and other yeasts in the environment [33]. The contact of these birds with humans, especially in urban environments, represents a risk to public health. The detection of multidrug-resistant yeasts in the gastrointestinal tract of synanthropic birds is also concerning, as these birds may be reservoirs for the transmission of drug-resistant fungal infections to humans [30].

Previous studies have reported direct transmission of fungal infections to poultry farmers and poultry keepers through contact with bird bodies and excreta. Additionally, the dimorphic fungus *Histoplasma capsulatum*, which can cause serious illness in immunocompromised people, has been found in the droppings and body parts of certain species of birds, including chickens [34,35]. However, the role of the yeast microbiota in birds and their propensity for pathogenicity and infection is not yet well established. Although it is known that birds can act as carriers of pathogenic fungi for humans, there is still a lack of clear evidence to indicate that domestic birds are reservoirs of yeast [2,36].

Blood infections caused by non-albicans *Candida* species have become increasingly common. Additionally, invasive infections with other rare yeasts, such as *Trichosporon* spp., *Geotrichum* spp., *Cryptococcus* spp. and *Rhodotorula* spp., have also been recently reported. Studies have suggested that gastrointestinal colonization may be a potential source of more serious fungal infections [30]. Laying hens carry yeasts, mainly *Candida* spp., in their cloaca and can contaminate the environment through their feces and eggs. The prevalence of yeast in samples of cloacal swabs and feces from chickens was higher than that found in other birds, indicating the role of laying hens as potential transporters and disseminators of these microorganisms. Feces showed a greater occurrence and population size of yeast compared to cloacal swab or egg samples, suggesting that feces are a suitable enrichment medium for yeast growth [31].

The presence of species such as *C. parapsilosis* and *R. rubra* in fecal and egg samples, in addition to the cloaca, suggests that laying hens can disseminate yeasts in the environment through their feces and eggs. Yeasts were also found on eggshells and in their yolks and whites, indicating that these microorganisms can contaminate the entire egg during passage through the cloaca or after oviposition [31]. Egg storage temperature has been shown to have a significant effect on the yeast population on the shell. Eggs stored at higher temperatures showed an increase in yeast prevalence and population size compared to those stored at lower temperatures. This suggests that egg protective factors lose their ability to inhibit the growth of microorganisms during storage at higher temperatures [31]. Eggs contaminated by pathogenic yeasts represent a potential risk to human health, especially for immunocompromised patients. In this sense, adequate management and sanitation measures must be implemented in poultry farms to reduce the contamination of eggs by pathogenic yeasts [31].

Also noteworthy is the organic dust present on poultry farms, which is formed by a complex mixture of particles that include feces, feed, feathers, mites, bacteria, fungi, and their spores, as well as biological toxins. Dust derived from poultry production systems can contain bacteria and fungi of plant and animal origin. Fungi such as *Acremonium*, *Alternaria*, *Aurobasidium*, *Aspergillus*, *Basidiospores*, *Cladosporium*, *Chrysosporium*, *Drechslera*, *Epicoccum*, *Eurotium*, *Fusarium*, *Geomyces*, *Mucor*, *Penicillium*, *Pithomyces*, *Rhizomucor*, *Scopulariopsis,* and *Ulocladium* have been reported as prevalent in bird dust. Many of these fungi are recognized as allergenic strains [28]. Furthermore, fungal secondary metabolites, such as aurofusarin, deoxynivalenol, zearalenone, and infectopiron, have been found, which may have cytotoxic effects [28].

Regarding worker health, the dust deposited, airborne microorganisms, fungal secondary metabolites, and odors present in the environment of poultry farms represent potential risks to the respiratory health of farm and poultry workers. Therefore, it is important to carry out respiratory medical assessments of workers and implement prevention measures, such as the appropriate use of respiratory protection devices [28]. Poultry litter, which is composed of a plant substrate, feces, feed waste, feathers, and agricultural inputs, has been widely used as an organic soil fertilizer throughout the world. This is due to its composition (organic matter, micro- and macronutrients for vegetable crops) and low cost, which contribute to increasing the quality and productivity of agricultural crops [3,31].

To ensure efficient production of broiler chickens, it is necessary to use an appropriate litter material that meets the requirements for chemical, physical, and microbiological characteristics [37]. Studies have shown that wood sawdust bedding is associated with improvements in bird performance. However, it is necessary to keep in mind that the bedding material used can harbor various pathogens, such as viruses, bacteria, parasites, and fungi [2,37]. The combination of different bedding materials, along with chicken droppings, feathers, and chemicals used during the production cycle, can influence the development of these pathogens [37].

Assessing microbiological contamination in poultry litter and poultry facility environments is crucial for ensuring the well-being of animals, workers, and consumers. Conducting studies to examine microbial growth and the presence of pathogens in these areas is essential to identify health risks and implement appropriate mitigation measures. Notably, poultry litter significantly contributes to the spread of fungal contamination in poultry facilities [9]. Moreover, the activity of spreading litter often exposes poultry workers to elevated levels of dust, along with fungi and their byproducts, including volatile organic compounds (VOCs) and mycotoxins [38]. After it is used and removed from poultry facilities, the litter, containing keratinous materials, is incorporated into agricultural soils. However, this practice may pose potential threats to the soil environment and the health of both humans and animals [39]. The annual disposal of millions of tons of feathers, common in poultry production, can negatively affect soil and human health. Recent studies have investigated the presence of these fungi in soils globally, due to their potential to infect humans and animals, awakening interest in dermatologists and mycologists [40].

### 3.3. Fungi in Poultry Feed

Fungi, known as mycoflora or molds, can be present in animal feed and pose a threat to the quality of the feed. They can cause several problems, such as decreased seed germination, unpleasant or musty odors, loss of dry matter and nutrients, clumping, formation of mycotoxins and, consequently, a reduction in the economic value of the feed [41]. There are two main groups of fungi that can be found on grains: field fungi, such as *Absidia* spp., *Alternaria* spp., *Chaetomium* spp., *Cladosporium* spp., *Diplodia* spp., *Phaeoramularia* spp., and *Rhizopus* spp., and storage fungi, such as *Drechslera* spp. (Helminthosporium) and *Fusarium* spp., which attack the grains or seeds of plants while they are still growing [7].

The presence of these fungi is influenced by climatic factors, including precipitation and temperature. Some, like *Aspergillus flavus*, can act as plant pathogens and storage fungi [7]. Mycoflora growth in crops is highly dependent on weather conditions, with fungal invasion intensifying when crops are stressed, such as during droughts or insect infestations. Field fungi require high moisture content and are susceptible to drying out after harvest. Although some mycelium may remain dormant in food after harvest, most die during storage or transport. These xerophilic fungi, rarely found in growing grains, include *Candida* spp., *Hansenula* spp., and *Penicillium* spp. These fungi can be disseminated by insects and mites that feed on cereals [7,41].

The same fungi responsible for contaminating grown and stored grains have also been identified in commercial pet foods and feed ingredients. Some studies have detected these fungi in poultry feed, fishmeal, and rabbit feed. Insect droppings, exuviates, and the ability of insects to penetrate the protective waxy layer of grains can alter the grain environment and promote fungal infestation. Fungi, such as *Aspergillus* spp., *Fusarium* spp., *Mucor* spp., and *Penicillium* spp. have also been isolated from poultry litter, although the exact source of exposure to these fungi is uncertain and may occur through spilled feed, insect vectors, or fungal spores surviving in the birds’ intestinal tract [7,8].

## 4. Poultry Litter as a Source of Fungi for Agricultural Soils

The growing demand for animal products has led to a significant increase in intensive livestock farming, creating an imbalance between the number of animals and the environment’s carrying capacity. Intensive production of poultry, swine, and dairy results in the generation and concentration of large volumes of animal waste [15]. Due to the macro and micronutrient content in these waste materials, they can be repurposed as organic fertilizers in agricultural crops. This contributes to reducing environmental impacts, such as waste accumulation in ponds and piles, which can harm soil, surface, and groundwater quality. This approach aligns with the growing need for environmental sustainability and natural resource conservation [7].

The use of animal waste represents an alternative fertilization method for agricultural crops, primarily due to its organic matter and nutrient content, especially nitrogen (N) and phosphorus (P) [8]. This allows for the recycling of nutrients within ecosystems, improving soil properties and promoting the growth of beneficial microorganisms. Additionally, this practice replaces mineral fertilizers, which are finite and non-renewable nutrient sources, resulting in reduced production costs and a more sustainable system. However, it is important to note that the application of these wastes is often done empirically, with producers applying doses without proper evaluation of their impact on soil fertility, as these wastes contain nutrients in disproportionate amounts compared to plant requirements [8].

Furthermore, animal wastes are often applied to the soil surface, leading to nutrient accumulation in the top 10 cm of the soil. This results in the saturation of adsorption sites and a reduction in the soil colloid’s nutrient retention capacity, particularly for phosphorus. The amount of organic waste to be applied in a specific area depends on various factors, such as waste composition, organic matter content, soil fertility, the nutritional needs of the cultivated crop, and regional climatic conditions [8]. Poultry litter consists of bird excrement, feathers, wasted feed, and the moisture-absorbing material used on the floor of poultry houses. It is a nutrient-rich waste, and its composition can vary based on the feed nature, the amount and type of floor covering material in the poultry house, the duration the birds spend on the material, the number of birds per area, and storage conditions. Nutrient levels can also fluctuate depending on the poultry litter’s origin and the number of layers of absorbent material (e.g., wood shavings) used [42,43].

While the benefits of using animal waste for fertilizing agricultural areas are clear, excessive use can harm productivity, exceeding soil’s carrying capacity and causing significant environmental contamination [37]. Therefore, continuous monitoring of soil quality, especially focusing on microbiological aspects, is essential to ensure the proper utilization of animal waste in agriculture. Poultry litter, commonly used as a substrate for poultry in agricultural systems, may contain fungal spores and microorganisms naturally present in poultry facilities. When this litter is disposed of in agricultural soils, it can release fungal spores into the environment, potentially introducing fungi into the soil [44].

This introduction of avian fungi into agricultural soil is a significant aspect with implications for biogeochemical cycles and soil microbiology [3]. Additionally, the extensive use of fungicides in agriculture to control fungal diseases in crops raises concerns about their entry and persistence in agricultural soils, which can have environmental impacts such as groundwater contamination and fungal resistance. Investigating the presence and effects of both poultry litter and fungicides in soils is crucial for sustainable agriculture management and mitigating adverse impacts [44].

Opportunistic fungal infections are a growing global health concern, and both domestic and wild birds may play a role as carriers of pathogenic fungi with potential impacts on human health [30]. Human exposure to pathogenic fungi like *Candida*, *Cryptococcus*, *Geotrichum*, *Rhodotorula*, *Saccharomyces*, and *Trichosporon* from avian sources poses significant health risks [30,45]. The potential transmission of zoonotic pathogens from synanthropic birds to humans is particularly alarming, given that these birds might harbor drug-resistant fungi [32].

Fungi, including *Aspergillus* spp., *Penicillium* spp., *Fusarium solani*, *Geotrichum candidum*, *Nannizzia gypsea*, *Rhizopus stolonifer*, *Trichoderma* spp., and *Trichophyton* mentagrophytes, are commonly found in soils fertilized with poultry litter. These fungi may present infection risks to humans, particularly in specific environmental conditions. Aspergillus species, abundant in such soils, can be pathogenic to humans when their spores are inhaled, leading to lung infections, especially in individuals with compromised immune systems. Fusarium solani, another prevalent fungus, can cause opportunistic infections affecting the skin, nails, eyes, and internal organs, notably in hospital settings. Although more common in the food industry, certain strains of *Geotrichum candidum* can infect humans, primarily in moist skin areas [39].

Dermatophytes like *Nannizzia gypsea* and *Trichophyton mentagrophytes* cause skin, hair, and nail infections, including ringworm. Some *Penicillium* species, although mostly harmless, can produce harmful mycotoxins, potentially impacting humans through spore inhalation or consumption of contaminated food. While *Rhizopus stolonifer* is not commonly linked to human infections, specific *Rhizopus* species can affect immunocompromised individuals, causing invasive fungal infections. *Trichoderma* spp., typically considered beneficial in agriculture and enzyme production, can become opportunistic pathogens in humans, particularly in those with weakened immune systems [39].

It is essential to consider the interaction between fungi introduced by poultry litter and the fungicides used in agriculture. Fungicides can affect the diversity and activity of fungi in the soil. Furthermore, it is crucial to investigate whether exposure to fungicides can result in fungal resistance in strains present in the litter or soil. This research can provide insights into the potential risks to the effectiveness of fungicides in agriculture.

## 5. Antifungals in Poultry Farming

In poultry farming, it is common to use antifungals to prevent and treat infections in the flock. There are different types of antifungals used in this context, each with its own form of administration and mechanism of action. Systemic antifungals are administered orally or injected and are absorbed by the body to treat internal fungal infections. Examples of these antifungals include itraconazole, fluconazole, voriconazole, and amphotericin B. They work by inhibiting the growth and reproduction of fungi, helping to fight infection [30]. Topical antifungals are applied externally to the skin, feathers or areas affected by fungal infections in birds. They can be found in the form of sprays, lotions, or ointments. Some common examples of topical antifungals are clotrimazole, miconazole, and nystatin. These medications act directly on the fungi, inhibiting their growth and relieving the symptoms of superficial fungal infections [46].

Propionic acid serves as a feed preservative, thwarting the proliferation of fungi and yeast in poultry feed. Additionally, environmental fungicides may be applied within the poultry facility environment to regulate the growth of fungi and yeast on various surfaces, such as floors, walls, and bedding material. This application aids in diminishing the birds’ exposure to these microorganisms. In cases of fungal infections on the birds’ skin or nails, topical treatments, such as ointments and antifungals, are suitable remedies [47,48,49].

It is important to note that the use of any pesticide, including fungicides, in poultry farming must strictly follow government regulations, manufacturer recommendations, and good agricultural practices. Furthermore, it is essential to consider safety measures to prevent contamination of poultry feed and ensure the safety of animals and consumers [47,48]. Antifungal pesticides in poultry farming are chemicals designed to prevent, control, or eradicate fungal infections in poultry and the facilities where they are kept. These products are used to protect bird health, improve performance, and ensure the quality of final products such as meat and eggs [50].

## 6. Fungicides in Crop Production

A significant concern that must be highlighted is the massive use of fungicides in agriculture on a global scale. According to data from the Food and Agriculture Organization of the United Nations (FAO), fungicides represent the second most used chemical group in agricultural practices, second only to herbicides. This statistic emphasizes the extent of fungicide use in crop protection around the world, which raises environmental and health concerns. Fungicides are often applied to control fungal diseases that affect crops, contributing to increased agricultural productivity [11,51].

However, this practice is not without challenges, as excessive and inappropriate application of fungicides can result in residues in soil and water, negatively impacting soil biodiversity and posing potential risks to human health. In the context of this study, it is important to highlight that soils that receive poultry litter may be subject to the massive entry of fungicides derived from plant agriculture [11]. This entry of fungicides into agricultural soils can occur in several ways, including contaminated water runoff and residues left on crops after application. This complex interaction between the introduction of fungi from poultry farming and exposure to agricultural fungicides in soils is an important and challenging research topic [44,52].

The Harmonized System (HS) classification groups disinfectants with various pesticide products, encompassing insecticides, rodenticides, fungicides, herbicides, anti-sprouting agents, plant-growth regulators, disinfectants, and similar items under subheading HSN Code 3808. Despite many of the traded disinfectants not being primarily intended for agricultural use, their substantial contribution to the increasing trade should be recognized. Over the last three decades, pesticide use has averaged 1.58 kg per hectare per year, 0.37 kg per person per year, and 0.79 kg per 1000 I$ per year [11].

In 2020, the Americas took the lead as the largest global importers of pesticides, receiving a total of 1.1 million tons valued at USD 6.9 billion, with a noteworthy 0.6 million tons of disinfectants, signifying a 160 percent increase from the previous year; fungicide usage increased from 93 to 177 kt. Meanwhile, Oceania had limited participation in the pesticide trade, with most exports remaining within the region. In the same year, the total imports within Oceania reached 342 kt, valued at USD 1.4 million, while fungicide usage increased from 3 to 5 kt [11].

Furthermore, Africa predominantly imports pesticides from countries outside the continent and tends to retain most exported pesticides within the region. In 2020, the region imported a total of 850 kt of pesticides (USD 3.1 million), with 779 kt (USD 2.8 million) originating from other global regions. The total pesticide exports amounted to 71 kt (USD 0.45 million), with only 20 kt being exported to non-African countries (USD 0.12 million). The usage of fungicides and bactericides (33 percent) and insecticides (27 percent) in total pesticide use has remained stable over the past decade [11].

Fungicides used in poultry farming can vary depending on specific needs and local regulations. The classes of fungicides most used in agriculture cover a wide range of compounds, each with their own mechanisms of action and specific applications. Among the most prominent, we find sulfur fungicides, effective as contact fungicides against powdery mildew and mildew; cupric fungicides, which include copper sulfate and are known for their action against fungal and bacterial diseases; dithiocarbamates, which inhibit the growth of fungi and are used in crops such as potatoes and tomatoes; isophthalonitriles, systemic fungicides used to control downy mildew in fruit crops; chloroaromatics, such as chlorothalonil, effective against various diseases; dicarboximides, with systemic properties and wide application in grain crops; and organostatics, such as benomyl, used both in agricultural crops and in gardens. Each of these classes plays a crucial role in managing fungal diseases in crops, ensuring the health and productivity of plantations [53].

Some fungicides can be used in poultry farming, such as Tebuconazole. Tebuconazole is a broad-spectrum fungicide used to control a variety of fungal diseases in grain and vegetable crops, which can be a source of food for birds. Azoxystrobin is used to control fungal diseases in agricultural crops, and its safe use around animals must be strictly regulated. Mancozeb is a fungicide used on various crops to control fungal diseases. If used near areas where birds feed, it must be done with caution to avoid direct exposure. Flutriafol is used to control various fungal diseases in crops such as corn and wheat. Cyproconazole can be used on crops to prevent fungal diseases. Use around birds must be done in accordance with regulations and with care to avoid direct contamination [49,50].

**Figure 1:**
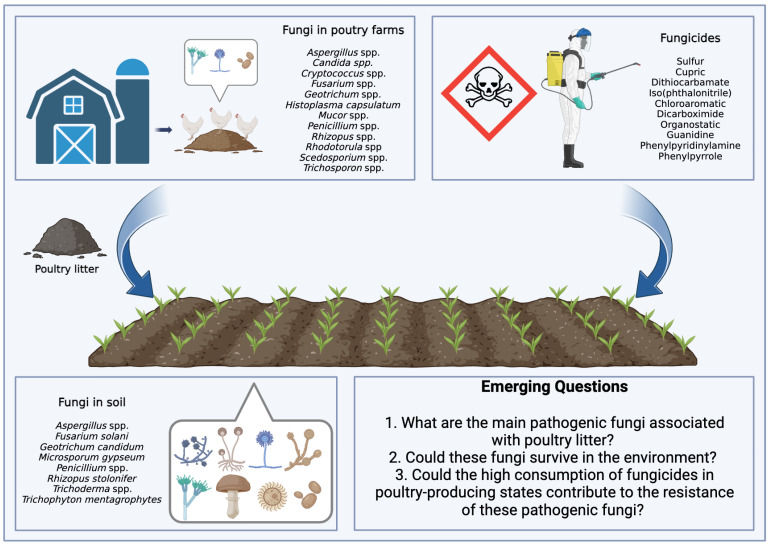
Cycle of contamination by fungi derived from poultry litter and fungicides applied to agricultural soils. Interactions that drive emerging questions.

Brazil plays an important role in global food production. Currently, there is a scenario of increased use of pesticides, with data from the Brazilian Institute of Environment and Renewable Natural Resources indicating a significant increase in the consumption of active ingredients. In 2016, the country used 541,861 tons of these ingredients, a number that grew to 719,507 tons in 2021. It is noteworthy that fungicides, belonging to the pesticide class, appear as the second most used category, with a notable increase of 66,222 tons in 2016 to 128,757 tons in 2021. In this context, two fungicides, mancozeb and chlorothalonil, occupied the positions of third and fourth most consumed pesticides in the country in 2021 [54]. Figure 1 illustrates the complex interaction between poultry litter application as fertilizer in agricultural soils and fungicides used in crop production, highlighting the challenges of managing soil fungal contamination and antifungal resistance.

### Antifungal Susceptibility Testing

The demand for antifungal susceptibility testing (AFST) is on the rise due to an increasing number of patients with risk factors for invasive fungal infections, widespread antifungal use, and the emergence of antifungal resistance. The primary objective of AFST is to ascertain Minimum Inhibitory Concentration (MIC) values, which serve as a guide for antifungal therapy and resistance monitoring. This testing is recommended when there are concerns about resistance, or when the treatment of invasive infections is ineffective [55].

While methods like broth microdilution according to the Clinical and Laboratory Standards Institute (CLSI) and the European Committee on Antimicrobial Susceptibility Testing (EUCAST) are considered gold standards, they are time-consuming [55]. Alternative methods, including disk diffusion, epsilometric testing, colorimetric broth microdilution, and automated spectrophotometers, are also available. Recently, simplified approaches, such as a novel four-well plate agar method for screening echinocandin susceptibility in *Aspergillus*, have been developed [55].

Additionally, MIC test strips and *Sensititre YeastOne* have shown substantial agreement with traditional broth microdilution methods. While AFST methods are invaluable, they have their limitations, such as delays in obtaining results and challenges in interpreting results for certain combinations of fungi and antifungals. Technical and fungal factors can also influence MIC readings. It is important to note that AFST cannot be applied to *Pneumocystis jirovecii* since this species does not grow in in vitro culture media [56].

There is a growing interest in culture-independent approaches, such as MALDI-TOF MS technology and molecular-based resistance detection, which can provide quicker results but may come with interpretation challenges. Although MALDI-TOF MS shows promise, it requires simplification and automation to cover a broad spectrum of fungi and antifungals effectively. Additionally, BRCAST (Brazilian Committee on Antimicrobial Susceptibility Testing) has been actively contributing to global efforts in standardizing and improving AFST practices [12,55].

## 7. Antimicrobial Resistance

Antimicrobial resistance (AMR) is a pressing global health concern, with the overuse of veterinary drugs, including antimicrobials in animal agriculture, contributing significantly to its emergence [4]. The excessive use of antimicrobials in animal feed production, primarily to promote growth and prevent diseases, is a major driver of AMR. This problem is expected to persist as agricultural practices expand in developing nations [57,58]. There is substantial knowledge about AMR in common foodborne zoonotic pathogens and organisms frequently found in birds [12,37,59,60].

The poultry industry is a cornerstone of global food production, notably chicken meat, which is widely consumed due to its low production costs and absence of cultural or religious constraints. Consequently, a wide range of antimicrobials are routinely administered to poultry worldwide, primarily through oral routes, to enhance productivity and protect bird health [16]. Remarkably, the World Health Organization (2017) classifies most of these antimicrobials as critically important for human medicine. The indiscriminate use of antimicrobials in poultry farming poses concerns related to the development of AMR in both commensal and pathogenic organisms. This has repercussions, including the presence of antimicrobial residues in poultry products like eggs and meat, with potential consequences for human health [3,15,16].

Poultry farms are environments that harbor a significant number of microorganisms, both pathogenic and non-pathogenic. The abundant presence of microbes in poultry farming facilities is a natural characteristic, given the interaction between birds, their environment, feed, water, and soil. However, the density of microorganisms and their diversity can be influenced by several factors, including management practices, nutrition, use of antimicrobials, and hygiene [15].

## 8. Azole Resistance

### 8.1. Azole Resistance in the GENUS CANDIDA

Antifungal resistance mechanisms vary between species and antifungal agents. The main determinants of azole resistance generally involve changes in drug target affinity, drug target overexpression, and efflux pumps (Table 1) [57]. This is particularly relevant for the treatment of invasive candidiasis in developing countries where azoles are frequently used. Although the modification of the drug target, ERG11, is an important resistance mechanism in some species, such as *C. albicans* and *C. auris*, it does not play a prominent role in *C. glabrata*. Overexpression of ERG11, controlled by UPC2, results in overexpression of the drug target [61].

Furthermore, overexpression of efflux pumps, regulated by transcription factors such as TAC1 and MRR1, is a common response to azole stress. The association between mutations that cause overexpression of these efflux pumps and resistance in *C. parapsilosis*, *C. tropicalis*, and *C. krusei* is still little studied [62]. Recent studies have identified mutations in UPC2, TAC1, and MRR1 in azole-resistant isolates of *C. tropicalis* and *C. parapsilosis*. GOF mutations in TAC1B play a prominent role in azole resistance in *C. auris*. In *C. glabrata*, GOF mutations throughout the PDR1 gene have been identified in azole-resistant isolates, and these mutations are associated with overexpression of efflux pumps and virulence [61,62,63].

Some adhesion-related genes, such as EPA3, are also involved in azole resistance in *C. glabrata*, which reduces the intracellular concentration of azole drugs in clinical isolates. Studying these mechanisms is crucial, especially in the context of the increasing prevalence of azole-resistance in the *Candida* genus in several countries. This research could have important implications for drug discovery and the development of effective treatments for infected patients [64,65,66,67,68,69].

### 8.2. Azole Resistance in A. fumigatus

Azole resistance in *A. fumigatus* is multifactorial and involves the modification and overexpression of the azole drug target, Cyp51A, as well as overexpression of efflux pumps, mainly Cdr1B and AtrF. One of the main factors in azole resistance is the occurrence of 34 and 46 bps tandem repeats (TR34 and TR46) upstream of Cyp51A, which serve as additional binding sites for regulatory proteins, such as SrbA and AtrR, resulting in the overexpression of Cyp51A and Cdr1B, respectively [68].

These TR duplications are often accompanied by mutations in Cyp51A, such as TR34/L98H and TR46/Y121F/T289A, which are common in azole-resistant isolates. Furthermore, CCAAT-binding elements such as HapB, HapC, and HapE negatively regulate Cyp51A expression, and strains containing HapEP88L are associated with azole resistance even without mutations in Cyp51A. Recent studies have also highlighted the role of cellular components and related pathways in azole resistance in *A. fumigatus*, resulting in significant advances in this area of research [61,68].

### 8.3. Echinocandin Resistance

Resistance to echinocandins in several *Candida* and *Aspergillus* species is mainly caused by mutations in specific regions (hotspot regions, HS) of the FKS genes, which encode the catalytic subunits of β-1,3-d-glucan synthase [69,70]. The prevalence of resistance is relatively low in *C. albicans*, but it is more significant in *C. glabrata*, *C. tropicalis*, *C. auris*, and *C. parapsilosis* [68]. In *C. glabrata*, the amino acid substitutions S629P/T and S663P/F/A in Fks1 and Fks2, respectively, are linked to resistance [69]. In *C. albicans*, mutations such as S456P/F and Ser641P/F are common [67]. *C. tropicalis* and *C. auris* have predominant mutations, such as S645P and S639P/F/Y, in Fks1 [62,70]. *C. parapsilosis* has intrinsic resistance due to a P660A polymorphism in Fks1 [62].

In *A. fumigatus*, resistance to echinocandins is rare, but the increasing use of these drugs due to resistance to triazoles has led to the emergence of isolated resistance. Mutations in FKS, such as F675S, are observed in some *A. fumigatus* isolates [71]. However, recent studies have also identified echinocandin-resistant isolates without mutations in the FKS gene, due to lipid changes in the enzymatic microenvironment of β-1,3-d-glucan synthase. Mutations outside the HS regions can also cause resistance, and occasionally, susceptible isolates harbor weak mutations in the HS regions, leading to therapeutic failure. Therefore, the combination of FKS gene sequencing and antifungal susceptibility testing (AFST) is essential for an accurate assessment of echinocandin resistance [67].

### 8.4. Antifungal Resistance in Cryptococcus spp. and Pneumocystis spp.

Antifungal resistance in *Cryptococcus* involves mutations in genes such as ERG11, as well as overexpression of ERG11 and the ATP-binding cassette (ABC) transporter AFR1, resulting in resistance to azoles, such as fluconazole [66]. Resistance to 5-fluorocytosine in *Cryptococcus* is associated with mutations in genes such as UXS1, FUR1, and FCY2, as well as alterations in capsule biosynthesis [72]. In *Pneumocystis jirovecii*, resistance to azoles has been associated with mutations in the ERG11 gene, although the lack of effective response to azoles may also be related to the production of ergosterol by *P. jirovecii* itself [73].

Moreover, mutations in the dihydrofolate reductase (DHFR) and dihydropteroate synthase (DHPS) genes lead to resistance against sulfa drugs, which in turn has implications for clinical outcomes. These outcomes encompass factors such as the duration of mechanical ventilation, mortality rates, and adverse reactions. Examining mutations in DHFR and DHPS proves valuable in the context of patient care, as it aids in preventing treatment inefficacy and life-threatening complications. However, it is worth noting that the prevalence of these mutations varies among diverse patient populations [66].

## 9. The Significance of the One Health Concept

The World Organization for Animal Health (WOAH), together with the World Health Organization (WHO) and the Food and Agriculture Organization of the United Nations (FAO), introduced the One Health concept in the early 2000s. This initiative emphasizes the interdependence between human health, animal health, and the ecosystems in which they coexist. Around 60% of pathogens capable of infecting humans originate from domestic or wild animals [2]. The importance of the interaction between environmental quality, animal, and human health highlights the need for investigations to understand the mechanisms of microbial transfer, both pathogenic and non-pathogenic, between humans and animals in ecosystems [36].

This transfer of microorganisms can occur between people and animals, mediated by the environment, including the influences of urbanization and environmental stress. These influences can lead to changes in microorganisms and the development of intrinsic virulence factors, with potential disastrous consequences for human health [74]. Increased consumption of foods of animal origin has driven the growth of livestock production, resulting in an increase in the distribution and intensity of parasitic infections, including zoonotic diseases [29].

In poultry farming, it is common to use confinement structures with a high density of birds, which presents challenges in maintaining ideal microclimate and animal hygiene conditions [31]. Poultry litter quality is critical to efficient poultry production, and different litter materials are associated with bird performance. The composition and age of animal litter, along with the environmental conditions of the poultry shed, can influence microbial counts. Furthermore, poultry litter may be an underestimated source of antimicrobial resistance transmission to animals, humans, and the environment, highlighting the importance of antimicrobial resistance surveillance in this context [3,31].

Microbial contamination in the poultry industry is a public health concern, and the “One Health” approach is necessary to consider human, animal, and environmental health. To assess microbial exposure in poultry facilities, appropriate sampling methods, such as air and surface sampling, along with laboratory analyses, are required [2]. The “One Health” approach has been adopted by several institutions and countries to monitor and evaluate zoonoses, antimicrobial resistance and foodborne outbreaks [2]. Furthermore, occupational exposure to microorganisms on poultry farms is an important and often overlooked concern. The assessment of biological risks and the protection of workers’ health are areas that need to be addressed more carefully. Employers are required to assess and prevent exposure to occupational hazards, including biological agents [2,37,46].

The One Health concept represents an integrated approach that recognizes the interconnectedness between human, animal, and environmental health. By applying this concept to poultry farming, especially in the control of antimicrobial resistance (AMR) and microbial contamination, it is possible to obtain substantial improvements in agricultural practices. A “One Health” approach emphasizes the need for cooperation between human health professionals, veterinarians, environmental scientists, and farmers to address complex problems.

1. *Integrated monitoring*: Implement integrated monitoring systems that track the spread of pathogens between humans, animals, and environments. This includes surveillance of resistant bacterial strains in poultry environments, as well as their potential transmission to humans.
2. *Responsible use of antifungals*: promote responsible use of antifungals in poultry farming by avoiding unnecessary administration and ensuring that medications are used in a prudent manner, under veterinary guidance.
3. *Proper waste management*: adopt sustainable waste management practices, such as adequate composting of poultry litter, to reduce the spread of pathogens and minimize environmental contamination.
4. *Education and awareness*: implement educational programs for farmers, poultry industry workers, and health professionals about the interconnection between human, animal, and environmental health, highlighting the importance of biosecurity.
5. *Collaborative research*: encourage interdisciplinary research that brings together experts in human, veterinary, and environmental medicine to address issues specific to poultry farming, such as identifying AMR factors and effective prevention methods.
6. *Quality standards*: develop and implement quality standards that address the presence of pathogens and antimicrobial resistance in poultry products, ensuring food safety and public health protection.

By adopting a “One Health” approach in poultry farming, it is possible to create synergies between different sectors, promoting global health, reducing antimicrobial resistance, and minimizing the risks of microbial contamination for the benefit of human, animal, and environmental health.

## 10. Future Perspectives

Poultry farming is a critical component of global food production, especially in addressing the growing demand for affordable animal proteins, primarily in developing nations. This age-old agricultural practice is vital for ensuring a consistent protein supply, contributing significantly to food security and nutrition, particularly in rural areas. However, it faces challenges related to environmental and public health [9,10].

Over the past two decades, global poultry production has experienced substantial growth, with this trend expected to continue, particularly when compared to pork and beef. Consumer preference for poultry is evident, but the intensive nature of this industry, characterized by large flocks raised in confined spaces with automated systems, raises concerns about parasitic infections and zoonotic diseases due to the high bird population density [1].

Proper management of poultry litter is crucial for maintaining bird health and crop quality when it is used as organic soil fertilizer. Nonetheless, concerns arise from the presence of pathogens, resistant microorganisms, heavy metals, and other contaminants in poultry litter, emphasizing the need for standardized guidelines and comprehensive studies, especially in developing nations, where data might be limited [3].

The remarkable diversity of fungi, estimated at 1.5 to 5 million species, underscores their pivotal roles in various ecosystems [5]. They contribute to organic matter decomposition, engage in mutualistic associations with plants, act as biocontrol agents, and contribute to soil stability. While most fungi offer benefits, some can be pathogenic to humans, leading to an increasing incidence of fungal diseases affecting millions of people. Fungi demonstrate their adaptability to environmental challenges, including climate change and increased pesticide resistance in agriculture. Understanding these adaptations is crucial for developing strategies to control fungal diseases and preserve ecosystems [5].

In the context of poultry farming, fungi actively participate in organic matter decomposition within poultry waste, playing essential roles in nutrient recycling and ecological balance. However, concerns arise from the potential of certain fungi to produce mycotoxins, which can be harmful when contaminating feed and food. The complex fungal microbiota in birds and their potential as sources of pathogenic microorganisms, including drug-resistant fungi, necessitate further research and investigation [19].

Addressing health risks associated with organic dust, including fungi and their spores, is crucial, particularly for poultry farm workers, necessitating health assessments and respiratory protection. The extensive use of poultry litter as a soil fertilizer highlights the importance of evaluating its chemical, physical, and microbiological characteristics to ensure its safety and efficacy [38]. Continuous monitoring is essential to mitigate health risks related to potential pathogen contamination in poultry litter, especially when it contains a mixture of bedding materials, poultry droppings, feathers, and chemicals [3].

Understanding how fungi interact with xenobiotics holds importance both for medical and environmental purposes [75]. Fungi’s ability to biodegrade chemicals offers opportunities for bioremediation, environmental protection, and potential applications in drug detoxification. Lastly, the increasing presence of veterinary drugs and pesticides in the environment through poultry litter is a significant concern, requiring ongoing monitoring and study, especially regarding its potential impact on soil and human health [76]. The utilization of fungi to biotransform and degrade pesticides offers an effective means of mitigating the environmental impact of these chemicals. Understanding the intricate interactions between fungi and xenobiotics is critical for multiple applications, ranging from drug production to environmental protection [75].

Looking ahead, addressing these challenges and advancing our comprehension of poultry farming and the kingdom of fungi necessitates interdisciplinary collaboration, data sharing, and a dedication to promoting the safety and sustainability of food production, environmental health, and public well-being [16]. The One Health concept, emphasizing the interdependence of human, animal, and environmental health, should guide future research and policies in this field, ensuring the health and safety of all stakeholders [3].

Fungal presence in poultry sheds can result in the development of fungal infections in birds, affecting their health and well-being. Moreover, concerns exist about the transmission of fungal pathogens from birds to humans, either through direct contact in poultry sheds or by consuming contaminated poultry products. Identifying these fungi is essential to prevent zoonotic diseases and develop control strategies. In addition to avian health issues, microbiological contamination in poultry facilities can pose risks to workers and consumers. Therefore, implementing biosafety practices is essential to minimize these risks [2,8].

Future research in poultry farming should emphasize understanding fungal ecology in agricultural and poultry environments, particularly those using poultry litter contaminated with veterinary drugs. Investigating fungal resistance to various antifungals and their enzymatic activity is crucial. Fungal studies in poultry farms are essential because these microorganisms directly impact bird health, poultry product quality, and food safety. Additionally, it is vital to explore the presence of fungi in agricultural and poultry environments, often fertilized with veterinary antimicrobial-contaminated poultry litter. This research is vital for comprehending the effects of these compounds on soil and crop fungal ecology and for identifying sustainable and eco-friendly practices. Reducing chemical product use and mitigating fungal risks in poultry farming is of utmost importance.

Another critical aspect is assessing fungal resistance to diverse antifungals, given the growing concern of drug resistance. Understanding how fungi develop this resistance is pivotal for shaping disease control strategies and selecting more effective treatments. Investigating fungal enzymatic activity is also significant, as these enzymes play roles in processes like organic material degradation, pathogenicity, and survival in challenging environments. Studying enzymatic activity provides insights into how fungi adapt and thrive in various surroundings, contributing to a broader understanding of fungal ecology, with potential implications for human health, agriculture, and environmental well-being. Evaluating fungal metabolic and structural adaptability is crucial, including assessing thermotolerance, resistance to oxidative stress, and enzymatic activities on solid media.

While this manuscript provides a comprehensive overview of global food security and environmental challenges associated with poultry farming, we recognize the importance of considering specific regional and national practices. Future research should focus on investigating how different regions are responding to these challenges, considering cultural, regulatory, and geographic particularities. By analyzing approaches adopted at regional and national scales, we can gain valuable insights into effective and adaptable strategies that can be implemented in different contexts.

Moreover, comparing traditional and organic poultry production systems can identify more sustainable practices and minimize fungal-related risks. Research on fungi in poultry farms aligns with the One Health concept, ensuring the well-being of humans, animals, and the environment while promoting agricultural sustainability. The knowledge generated by this study can serve as a solid foundation for future research in the field and as a reference for researchers investigating fungal ecology in similar environments.

*Emerging Questions:*

1. What are the main pathogenic fungi associated with poultry litter? Poultry litter is a suitable environment for the growth of fungi, and some may be pathogenic to birds. Identifying the main pathogenic fungi is essential to mitigate risks to bird health.

2. Could these fungi survive in the environment? The survival of pathogenic fungi in poultry litter and other agricultural environments is an important concern. This can affect the health of birds and the quality of agricultural products.

3. Could the high consumption of fungicides in poultry-producing states contribute to the resistance of these pathogenic fungi? The intensive use of fungicides in areas with greater poultry production may be associated with the development of resistance by pathogenic fungi, which would be a concern for both agriculture and poultry farming.

The challenge lies in finding a balance between crop protection and bird health, as well as well as the environment, and this requires careful analysis and effective mitigating measures.

### 10.1. Possible Specific Agricultural Management and Biosecurity Measures

Integrating specific agricultural management and biosecurity measures is crucial to addressing the challenges associated with the combined application of fungicides and poultry litter in agricultural areas. These measures are designed to promote sustainable farming practices, mitigate environmental risks, and safeguard public health. Crop rotation emerges as a key strategy, offering benefits such as reducing soil-specific disease pressure and enhancing overall soil health. Sustainable use guidelines for poultry litter, encompassing considerations of quantity, application frequency, and integration with other agricultural practices, can ensure responsible and eco-friendly utilization. Establishing monitoring and diagnostic programs facilitates early identification of potential fungal disease issues, enabling timely intervention. Conservation agriculture techniques, notably no-till practices, minimize soil disturbance, conserve moisture, and maintain soil structure. Responsible fungicide use, integrated pest management approaches, farmer training on good agricultural practices, and proper poultry litter disposal guidelines collectively contribute to a holistic strategy. Emphasizing continuous research is essential to develop disease-resistant crop varieties and assess the long-term impacts of these practices on soil and water. This comprehensive approach seeks to harmonize the imperative for increased agricultural productivity with sustainability goals, thereby minimizing adverse environmental and health consequences.

Some possible specific agricultural management and biosecurity measures include:

1. *Crop rotation*: implement crop rotation practices to reduce soil-specific disease pressure and improve soil health.
2. *Sustainable use of poultry litter*: develop guidelines for the sustainable use of poultry litter, considering the quantity applied, the frequency of application, and its integration with other agricultural practices.
3. *Monitoring and diagnosis*: establish monitoring and diagnostic programs to evaluate plant health regularly, identifying possible problems related to fungal diseases early.
4. *Conservation agriculture techniques*: promote conservation agriculture techniques, such as no-till, to minimize soil disturbance and promote conservation of soil moisture and structure.
5. *Responsible use of fungicides*: educate farmers on the responsible use of fungicides, including appropriate selection, correct dosage, and consideration of withdrawal periods.
6. *Integrated pest management*: encourage the implementation of integrated pest management approaches, which combine the use of fungicides with biological, cultural, and mechanical control practices.
7. *Farmer training*: provide regular training to farmers on good agricultural practices, including preventive measures to reduce the incidence of diseases.
8. *Proper disposal of poultry litter*: establish guidelines for the proper disposal of poultry litter, considering safe composting and minimizing environmental impact.
9. *Continuous research*: promote ongoing research to develop new disease-resistant crop varieties and to evaluate the long-term impact of using poultry litter and fungicides on soil and water. These measures aim to balance the need to maximize agricultural production with promoting sustainability and minimizing environmental and health impacts.

### 10.2. Xenobiotics and Fungi in Poultry Production

Xenobiotics are substances foreign to the body, that is, they are chemical compounds that are not naturally found in the human body or in other organisms. They include a wide variety of substances such as industrial chemicals, medicines, environmental pollutants, and many other compounds. When xenobiotics enter the body, they can trigger a series of biological responses, since the organism has not evolved to deal with these substances efficiently [77].

Prolonged exposure to these substances can lead to significant health risks. Our increasing reliance on xenobiotics to stimulate economic growth, ensure food production, and enhance health has raised serious concerns about environmental pollution. Many of these compounds do not naturally break down in the environment and are not effectively eliminated in wastewater treatment facilities [78]. While various physicochemical methods have been developed to address xenobiotic pollution, such as adsorption, precipitation, chemical oxidation, and membrane separation, biological approaches utilizing plants and microorganisms are considered more sustainable [79]. Consequently, there is substantial research effort focused on sustainable xenobiotic production and degradation [80].

Fungi, renowned for their role in decomposing organic matter, have garnered extensive attention due to their bioremediation potential. They possess a range of enzymes that facilitate the transformation of diverse compounds [75]. Industrial compound production often involves the use of hazardous reagents, high temperatures, and pressures, resulting in a substantial environmental impact [78]. Conversely, enzymatic reactions occur under gentle pH and temperature conditions, obviating the necessity for hazardous reagents. This establishes biocatalysis as an attractive substitute for conventional chemical synthesis. Fungal enzymes, specializing in oxidative reactions, hold particular significance for the eco-friendly manufacturing of valuable commercial compounds, rendering them a promising avenue for sustainable production [75].

Fungi could biodegrade xenobiotics, that is, break down these chemical compounds into simpler components. This property has been exploited in bioremediation applications, where fungi are used to remove pollutants from the environment. Genetically modified fungi can be used as bioreactors to produce compounds of interest, such as medicines or industrial chemicals, from xenobiotics. This is done by introducing specific genes into fungi so that they can synthesize the desired substances from xenobiotic compounds [75]. Some xenobiotics can be toxic to fungi, affecting their growth and metabolism. This can have significant consequences in ecosystems where fungi play a key role. In medicine, it is important to understand how xenobiotics, like medications, can interact with fungi in the human body. This can affect the effectiveness of medications or lead to unwanted side effects. Some fungi produce mycotoxins, which are toxic substances that can contaminate food and cause health problems in humans and animals when ingested. These mycotoxins are considered xenobiotics and represent a significant food safety challenge [75,78].

### 10.3. Xenobiotics Catabolized by Fungi

Fungi, such as *Penicillium chrysogenum*, *Aspergillus niger*, and *Cunninghamella bainieri*, have the capability to hydroxylate various model aromatic compounds, including acetanilide, acronicin, coumarin, and naphthalene. These findings suggest that fungi could serve as models for xenobiotic metabolism in mammals and could potentially be used for the large-scale production of important metabolites. The genera *Cunninghamella*, *Mucor*, *Aspergillus*, and *Fusarium* possess enzymes such as cytochromes P450 (CYPs), sulfotransferases, and glycosyltransferases, which catalyze phase I (oxidative) and phase II (conjugative) reactions, similar to the drug detoxification process in humans [75].

Traditional experiments involving the fungal biotransformation of drugs typically entail growing fungi in standard growth media, such as Sabauroud dextrose or potato dextrose. Cultures are then incubated with the target drug, and the resulting metabolites are extracted from the culture medium using appropriate organic solvents. Analytical techniques, such as gas chromatography (GC), liquid chromatography (LC), mass spectrometry (MS), and nuclear magnetic resonance spectroscopy (NMR), are used for analysis. Researchers often conduct comparative studies with human liver microsomes to identify similarities and differences between metabolites produced by fungi and those generated by humans [75].

An intriguing example is a study by Gaunitz et al. (2019), which compared the phase I metabolism of synthetic cannabinoids EG-018 and its fluorinated analog EG-2201 using human liver microsomes, CYP isoenzymes, and *Cunninghamella elegans*. The results indicated a correlation between fungal metabolites and those produced by liver microsomes, showing that fungi not only mimic mammalian enzymes but also have the capacity to biotransform fluorinated substances. Given the prevalence of fluorinated anthropogenic compounds, the role of fungi in their biotransformation holds significant relevance [81].

Furthermore, fungal nonspecific peroxygenases (UPOs), which are related to CYPs, have been identified as effective in transforming drugs into mammalian-like metabolites. The implementation of Metabolites in Safety Testing (MIST) regulations by the Food and Drug Administration (FDA) has made access to these metabolites crucial for toxicity evaluation. Fungi are easily scalable microorganisms, providing a sustainable alternative to chemical synthesis for drug metabolite production [82].

Another key reason for investigating medicine biotransformation by fungi is the potential to produce metabolites distinct from those observed in mammals. These metabolites may exhibit modified bioactivity and serve as potential leads for new medicines. *Cephalosporium aphidicola* and *Fusarium lini* have been utilized to biotransform the anticancer steroid drostanolone enanthate, resulting in the production of eight different products through deesterification, carbonyl reduction, dehydrogenation, and hydroxylation. Five of these metabolites were previously unknown and exhibited higher activity against certain cell lines compared to the original drug [83].

Moreover, research has explored how fungal biofilms can be applied to enhance drug metabolite production. Biofilms provide mechanical stability and resistance to toxic compounds, naturally immobilizing fungi. This has the potential for semi-continuous drug metabolite production, making fungi an attractive option for such applications. In the context of pesticide degradation, microorganisms, including fungi, have been extensively studied for their ability to break down pesticides. Many of the fungi mentioned earlier are also frequently utilized in pesticide biotransformation and biodegradation studies [84].

*Cunninghamella elegans* has been employed to degrade various pesticides, including pyrethroids and organophosphate pesticides [85]. *Aspergillus sydowii* and *Penicillium decaturense* have demonstrated the ability to degrade the organophosphate pesticide methyl parathion, converting it into p-nitrophenol [86]. Furthermore, *Aspergillus niveus*, *Aspergillus terreus*, and *Cladosporium cladosporioides* have successfully detoxified 3,4-dinitroaniline, a pollutant resulting from phenylurea herbicides [87]. These examples highlight the significant role of fungi in the biotransformation and degradation of pesticides, emphasizing the relevance of fungal strategies in mitigating the environmental impact of these compounds.

## 11. Conclusions

Poultry farming plays a crucial role in global food production, but it faces environmental and public health challenges, particularly in developing countries. Fungi in poultry litter contribute to organic matter decomposition, nutrient recycling, and ecological balance but can also pose risks due to the potential production of mycotoxins and pathogenic microorganisms, including drug-resistant fungi. Health risks associated with organic dust, including fungi, should be addressed through health assessments and respiratory protection for poultry farm workers. The application of poultry litter and fungicides to agricultural soils raises emerging questions about the potential selective pressure for the emergence of resistant fungal pathogens in food production areas. Understanding the interactions between fungi and xenobiotics is crucial for applications in bioremediation, environmental protection, and drug detoxification. Interdisciplinary collaboration and the One Health concept are essential for advancing research in poultry farming, ensuring food safety, environmental health, and public well-being.

It is vital to point out future research directions that can provide significant advances in mitigating environmental risks associated with the combined use of fungicides and poultry litter. A promising line of research consists of exploring alternative agricultural practices, such as the adoption of agroecological systems, crop rotation, and organic agriculture. These approaches aim to reduce dependence on chemical inputs and promote soil health in a sustainable way. Furthermore, an in-depth analysis of emerging technologies, such as remote sensing techniques and predictive modeling, can offer valuable insights for efficient agricultural waste management. Future research could also focus on evaluating specific management practices, agricultural regulations, and awareness strategies to mitigate identified risks. By directing efforts toward these research directions, we can move toward more sustainable and safe agricultural practices, minimizing adverse impacts on human and environmental health.

## Figures and Tables

**Table 1:** Species/genus, associated resistance mechanisms, and the relevant references for each of the mentioned fungal species.

Species/Genus	Drug Class	Associated Resistance Mechanisms	Reference
*Candida* spp.	Azoles	Changes in drug target affinity, drug target overexpression, overexpression of efflux pumps	[57,61,62,63,64]
*A. fumigatus*	Azoles	Modification and overexpression of Cyp51A, overexpression of efflux pumps	[58,65]
*Candida* and *Aspergillus* spp.	Echinocandins	Mutations in FKS genes (hotspot regions)	[62,66,67,68,69,70,71]
*Cryptococcus* spp.	Azoles	Mutations in ERG11, overexpression of ERG11, ABC transporter AFR1	[66,72]
*Cryptococcus* spp.	Pyrimidines	Mutations in genes such as UXS1, FUR1 and FCY2, as well as alterations in capsule biosynthesis	[72]
*Pneumocystis* spp.	Azoles	Mutations in ERG11, dihydrofolate reductase (DHFR), dihydropteroate synthase (DHPS) genes	[66,73]

## Data Availability

Not applicable.
